# Noncoding RNA control of neuroinflammation in Alzheimer’s disease: from immune cell states to precision therapeutics

**DOI:** 10.3389/fimmu.2026.1998588

**Published:** 2026-10-28

**Authors:** Shengqiang Zhou, Yanjun Chen, Xiping Yang, Liqin Xiang, Bo Li, Lingling Qiu, Zhuo Liu, Tiantian Zhang, Fang Liu, Liuting Zeng, Guo Mao

**Affiliations:** 1 Hunan Provincial Hospital of Integrated Traditional Chinese and Western Medicine (The Affiliated Hospital of Hunan Academy of Traditional Chinese Medicine), Changsha, Hunan, China; 2 Hunan University of Chinese Medicine, Changsha, Hunan, China; 3 The First Hospital of Hunan University of Chinese Medicine, Changsha, Hunan, China; 4 Mucosal Immunology Section, National Institute of Dental and Craniofacial Research (NIDCR), National Institutes of Health (NIH), Bethesda, MD, United States

**Keywords:** Alzheimer’s disease, circular RNA, extracellular vesicles, long noncoding RNA, microglia, microRNA, neuroinflammation, RNA therapeutics

## Abstract

Neuroinflammation is an active and heterogeneous component of Alzheimer’s disease, shaped by dynamic interactions among microglia, astrocytes, neurons, and brain-interface compartments. Noncoding RNAs, including microRNAs, long noncoding RNAs, circular RNAs, and emerging small-RNA species, regulate innate immune sensing, interferon responses, phagocytosis, metabolic adaptation, inflammasome activity, and neuronal stress. Their effects are strongly context dependent: the same noncoding RNA can exert different or even opposing functions according to cell identity, intracellular or extracellular localization, disease stage, and dose. Extracellular vesicles and extracellular RNA sensing further extend noncoding RNA activity beyond cell-autonomous regulation, creating intercellular circuits that couple neuronal stress to glial immune states. Noncoding RNA-regulated inflammation intersects with amyloid-β production and clearance, tau-associated stress, synaptic dysfunction, mitochondrial impairment, and regulated cell death. Rather than acting as isolated inflammatory switches, noncoding RNAs appear to tune the strength of pathological feedback networks that determine whether proteotoxic and inflammatory stress is contained or progressively amplified. The translational value of individual noncoding RNAs depends on the convergence of causal evidence, mechanistic resolution, human relevance, and therapeutic tractability. Human brain, cerebrospinal fluid, patient-derived extracellular vesicles, and induced pluripotent stem cell models are beginning to bridge experimental mechanism and human disease, while antisense oligonucleotides, engineered extracellular vesicles, targeted nanoparticles, and synthetic circular RNAs provide emerging therapeutic routes. Pleiotropy, cell-type specificity, disease-stage dependence, endosomal trafficking, and innate RNA sensing remain major barriers. Context-resolved noncoding RNA regulatory nodes that control defined neuroimmune states may ultimately enable precision neuroimmune reprogramming while preserving protective immune functions.

## 1 Introduction

Alzheimer’s disease (AD) is a multicellular, temporally evolving disorder in which amyloid-β (Aβ) and tau pathology interact with innate immunity, glial function, vascular homeostasis and neuronal resilience. Neuroinflammation is therefore not adequately described as a terminal reaction to neurodegeneration. Genetic, neuropathological and single-cell data instead place immune processes within disease progression, with microglia embedded in a broader ecosystem of astrocytes, neurons, vascular cells, brain-interface tissues and peripheral immunity (1–3). These responses are neither uniformly harmful nor static: programs that initially support containment, phagocytosis or tissue adaptation can become metabolically constrained or neurotoxic as pathology accumulates.

This state-based view changes how noncoding RNA (ncRNA) biology should be interpreted. miR-155 participates in interferon-gamma (IFNγ)-dependent microglial transitions and phagocytosis, yet complete microglial deletion can reduce amyloid pathology while provoking hyperexcitability and seizures (4, 5). The miR-25802–Krüppel-like factor 4 (KLF4)–nuclear factor kappa B (NF-κB) axis links RNA perturbation, target rescue, inflammation, amyloid pathology and cognition within a single causal chain (6). These findings favor state-transition control over fixed inflammatory identities.

ncRNA action also extends well beyond canonical miRNA repression. Circular RNA Dlg1 (circDlg1) controls phosphodiesterase 4B (PDE4B) protein stability, whereas the SNHG3–miR-128-3p–UBR5 network couples inflammatory and oxidative-stress phenotypes to AD-related outcomes (7, 8). Across cells, patient-derived neuronal and microglial extracellular vesicles (EVs) carry miRNAs that alter recipient-cell immunity. Extracellular miR-29a-5p activates mouse TLR7 and human TLR7/8 in receptor-reporter systems; however, the downstream microglial inflammatory and neuronal-injury phenotypes demonstrated in the cited experimental models were primarily TLR7-dependent (9–11). Mechanism therefore depends on both molecular architecture and compartment.

This mechanistic breadth creates both therapeutic opportunity and liability. Antisense inhibition of miR-485-3p improves Aβ, tau, inflammatory and cognitive phenotypes while the candidate is detectable in human brain, cerebrospinal fluid (CSF) and plasma (12); engineered circular RNA aptamers show that RNA can also be designed as a therapeutic material (13). An Author Correction to the latter study was published on 12 August 2026; the present review interprets the circular-RNA platform using the corrected figures and source data. Yet pleiotropy makes dose, cell specificity and disease stage integral to efficacy and safety.

Taken together, the literature places ncRNAs within a neuroimmune system organized by cell state, molecular mechanism, intercellular communication, and pathological feedback, while causal strength, human relevance, and therapeutic tractability remain distinct dimensions of evidence. Rather than cataloguing ncRNAs by RNA class or assigning them fixed pro- or anti-inflammatory labels, this review integrates four axes that are often considered separately: neuroimmune cell state, compartment-specific mechanism, multidimensional strength of evidence, and translational tractability. Apparent discordances across studies are used as tests of context dependence rather than averaged into a single direction of effect.

### 1.1 Literature identification and evidence appraisal

During preparation and revision of this narrative review, the literature base was updated through targeted searches of PubMed/MEDLINE and Google Scholar from database inception through 30 September 2026. Search combinations included ‘Alzheimer disease’ or ‘Alzheimer’s disease’ with ‘noncoding RNA’, ‘microRNA/miRNA’, ‘long noncoding RNA/lncRNA’, ‘circular RNA/circRNA’, ‘extracellular vesicle’, ‘microglia’, ‘astrocyte’, ‘neuroinflammation’, ‘innate immunity’, ‘TLR’, ‘inflammasome’, ‘amyloid’, ‘tau’ and ‘RNA therapeutics’. Reference lists of key primary studies and recent reviews were also cross-checked for relevant mechanistic reports. Priority was given to peer-reviewed primary studies that resolved cell or compartment, used selective RNA perturbation, downstream mediation/rescue, disease-relevant *in vivo* models, human-derived material or explicit therapeutic delivery/target-engagement experiments. Environmental exposure, injury and other AD-like models were retained when they clarified mechanism but are identified as indirect evidence rather than weighted as direct evidence for sporadic AD. Because the aim is mechanistic synthesis rather than exhaustive effect estimation, this is a structured narrative review rather than a systematic review; no PRISMA flow or meta-analysis was performed.

## 2 Principles of ncRNA-regulated neuroinflammation in AD

### 2.1 Neuroimmune cell states and context dependence

‘Microglial activation’ compresses a multidimensional process into a single label. Microglia integrate Aβ, damaged-cell products, extracellular nucleic acids, lipids, cytokines, complement and systemic signals, with distinct consequences for transcription, metabolism, phagocytosis, lysosomal function, migration and secretion. Contemporary neuroimmunology therefore favors heterogeneous, interconvertible states over a uniform resting-to-activated transition (1–4, 6).

Cell state, state transition, and functional program are preferable to M1/M2 polarization as descriptions of AD microglia. Historical studies that used M1/M2 terminology remain informative when interpreted through defined outputs such as inflammatory signaling, Aβ handling, redox balance, metabolic competence, or neuronal support rather than categorical identities. Accordingly, where an original study uses M1/M2 labels, we retain them only as study-reported terminology and reinterpret the results in terms of the measured functional program rather than as fixed microglial or astrocytic identities.

### 2.2 Molecular and compartmental logic of ncRNA regulation

Five mechanistic architectures recur across the evidence base. Canonical miRNA–mRNA repression modifies signaling hubs such as KLF4; lncRNA–miRNA–mRNA and circRNA–miRNA networks can alter miRNA availability, although competing endogenous RNA (ceRNA) claims require quantitative validation; RNA–protein interactions can control molecular stability, as in circDlg1–PDE4B; nuclear and epitranscriptomic mechanisms alter chromatin or RNA fate; and extracellular RNA can be selectively packaged, transferred or sensed by innate receptors (6–8, 11).

These architectures overlap: one ncRNA can use different mechanisms in different compartments, whereas distinct RNA classes can converge on the same inflammatory program. Interpretation therefore requires RNA identity, mechanism, cell type, compartment and disease state to be considered together.

An intracellular miRNA in an Argonaute complex is not biologically equivalent to the same sequence in an EV or extracellular fluid. Intracellularly, miRNAs predominantly regulate target transcripts; extracellularly, RNA can be protected, transferred to another cell or detected by Toll-like receptors (TLRs) and other nucleic-acid sensors (9–11). Compartment is therefore part of mechanism—and, for therapeutic RNA, part of safety.

### 2.3 Evidence, context, and causality

Differential expression identifies candidates but does not establish pathogenic function. Evidence strength is multidimensional: causal perturbation, mechanistic resolution, human relevance, and translational maturity answer different questions. A cell-specific genetic study may establish causality without a single closed target pathway, whereas a CSF signature may be clinically relevant without proving mechanism. Confidence increases when these dimensions converge (5–7).

Apparent contradictions should first be tested against differences in cell identity, intracellular versus extracellular localization, disease stage, inflammatory history, RNA dose, carrier, and functional endpoint. This principle is especially relevant to miR-155, miR-146a, miR-34a, miR-29a, and miR-25802, for which divergent effects have been reported across distinct experimental contexts. Such variables are mechanistically plausible explanations for discordance, but they should not be presented as demonstrated explanations unless directly tested within a common experimental framework.

## 3 ncRNA control of neuroimmune cell states

### 3.1 Microglial programs

Microglia provide the deepest cellular evidence linking ncRNAs to AD neuroinflammation. The literature spans innate sensing, interferon responses, Aβ handling, inflammasome activity, redox control, immunometabolism and structural remodeling. These outputs are coupled rather than modular: one ncRNA perturbation can simultaneously alter inflammatory tone, proteostasis, neuronal function and cognition.

NF-κB-centered pathways are a recurrent interface between ncRNAs and microglial inflammation. The miR-25802–KLF4–NF-κB axis provides comparatively strong causal and rescue evidence in microglial and *in vivo* AD models (6), whereas NEAT1–miR-361-3p–TNF receptor-associated factor 2 (TRAF2) and miR-107-5p/TLR4-related mechanisms reach the same inflammatory hub through different architectures (14, 15). Sex-dependent miRNA regulation of microglial fatty-acid synthesis further links inflammatory signaling to metabolic state (16). At the receptor level, extracellular miR-29a-5p activates mouse TLR7 and human TLR7/8 in reporter assays, whereas the functional microglial and *in vivo* neuroinflammatory effects in the cited study were demonstrated primarily through TLR7 (11). Acyloxyacyl hydrolase (AOAH)/miR-450b-5p findings in post-mortem human AD brain connect ncRNA regulation to sensitivity to microbial inflammatory products (17). Rather than merely tuning cytokine abundance, these ncRNAs appear to set the threshold and persistence with which microglia interpret innate immune stimuli.

Interferon-responsive states expose the limits of one-dimensional ‘pro-inflammatory’ labels. Type I interferon can drive neuroinflammation and synaptic loss in AD-related systems (18), yet miR-34a deficiency enhances nucleic-acid sensing and interferon-responsive microglial programs while improving selected AD phenotypes (19). Together with miR-155-dependent IFNγ state transitions (4), these findings indicate that the consequence of interferon signaling depends on the state produced, not simply its magnitude.

ncRNA control of microglial inflammasome and pyroptotic programs is mechanistically plausible but less mature than the NF-κB and Aβ-handling literature. miR-22 provides a principal anchor linking RNA regulation to inflammasome-associated injury and disease-level phenotypes (20). Much of the broader pyroptosis evidence, however, derives from neurons, astrocytes or therapeutic EVs, arguing for restraint when assigning a microglia-specific mechanism.

ncRNAs also couple inflammatory state to metabolic competence. Multi-omics analysis identifies a miR-28a-5p–SerpinA3N–PI3K/AKT axis linked to microglial inflammation and cognitive decline (21); tRF(Ala-AGC-3-M8) extends this control to extracellular signal-regulated kinase (ERK)/70-kDa ribosomal protein S6 kinase (p70S6K) signaling (22). miR-191-5p, let-7d-5p, miR-107-5p and additional lncRNA/circRNA pathways converge on MAPK, NF-κB and oxidative-stress programs (23–28). These data support an immunometabolic loop in which inflammatory signaling erodes metabolic competence and metabolic stress, in turn, reinforces inflammation.

Microglial state is also executed physically. Tincr links myosin phosphatase target subunit 1 (MYPT1)–non-muscle myosin IIA signaling to structural behavior and cognition (29), while circDlg1 connects RNA-dependent protein stability to microglial signaling (7). Migration, process dynamics and engulfment should therefore be treated as state variables alongside cytokine expression.

The clearest intersection between ncRNA-controlled inflammation and canonical AD pathology lies in microglial Aβ handling. RN7SL1-associated programs vary across vulnerable hippocampal–entorhinal regions (30); miR-146a and miR-181c-5p couple inflammatory state to Aβ-associated function (31, 32); and circDlg1 and miR-25802 provide stronger mechanistic links between microglial signaling and amyloid outcomes (6, 7).

A broader supporting literature implicates XIST, MAGI2-AS3, PRR34-AS1, miR-548m, miR-206, miR-199a-3p and miR-25-3p in Aβ-associated microglial injury or inflammatory responses (32–42). These studies expand the regulatory space, but their weight depends on model relevance and mechanistic closure. Aβ uptake should not be treated as synonymous with clearance. Recognition, internalization, lysosomal degradation and disposal are separable processes; ncRNA studies that collapse them into plaque burden risk misclassifying dysfunctional phagocytosis as therapeutic benefit.

Human disease-relevant systems are beginning to narrow the species gap. miR-132 modifies inflammatory responses in iPSC-derived microglia from patients with AD (43), while circDlg1, Tincr and RN7SL1 link experimental mechanisms to human disease-associated tissue (7, 29, 30). These models remain imperfect—iPSC reprogramming incompletely preserves aging and post-mortem tissue is weighted toward late disease—but they provide a stronger bridge to human causality than immortalized cell lines.

The microglial literature therefore argues against a single beneficial–harmful axis. Complete miR-155 deletion can lower amyloid burden yet cause hyperexcitability, seizures and excess mortality (5), whereas miR-155 also supports IFNγ-dependent state transitions and phagocytic programs (4). Therapeutic success will require selective rebalancing of pathological programs while preserving surveillance, clearance and network stability—not global suppression of ‘activation’.

### 3.2 Astrocytic programs

Astrocytic ncRNA evidence is smaller but mechanistically coherent, centering on inflammasome competence, chromatin regulation, glutamate homeostasis, redox control and neuron–glia signaling.

The SNHG14–miR-223-3p axis targeting NLR family pyrin domain containing 3 (NLRP3) is a leading astrocyte-centered mechanism (44), complemented by miR-34a/HDAC, miR-224-5p/NLRP3 and miR-146a/NF-κB pathways (45–47). Together, they place ncRNAs upstream of both inflammatory transcription and inflammasome readiness, while also underscoring cell-type dependence: the direction of miR-34a biology in astrocytes cannot be extrapolated to microglia.

Astrocytic ncRNAs can alter neuronal vulnerability without large shifts in canonical cytokines. interleukin-1 beta (IL-1β) regulates glutamate transporter 1 (GLT-1) through miR-181a, directly linking inflammation to glutamate homeostasis (48); the miR-10b-3p–NADPH oxidase 4 (NOX4) axis links astrocytic redox state to neuronal ferroptosis (49). These mechanisms broaden ‘neuroinflammation’ from cytokine output to the homeostatic functions that determine neuronal excitability and survival.

A miR-96-5p–cathepsin B pathway provides a particularly informative cross-cell example: neuronal inflammatory signals are interpreted through an astrocytic ncRNA program, and pathway manipulation alters astrocyte reactivity and memory (50). The mechanism is inherently cross-cellular and cannot be assigned to astrocytes alone.

### 3.3 Neuronal programs

Neurons are not merely downstream victims of glial inflammation. Their ncRNAs regulate inflammatory stress, neuritic integrity and cell-death susceptibility, while stressed neurons export RNA signals that reshape glial immunity.

A broad set of neuronal miRNAs converges on inflammatory signaling, apoptosis and neuritic integrity, including miR-125b-, miR-134-, miR-137-, miR-216a-5p- and miR-4284-centered pathways (51–55). LncRNA and circRNA mechanisms extend this network through CircUCK2, XIST, NEAT1, MALAT1, BDNF-AS, CircLPAR1 and circ_0000950 (56–64). Despite their molecular diversity, these axes repeatedly converge on a small number of functional outputs: inflammatory signaling, oxidative stress, apoptosis, neurite integrity and Aβ-associated injury.

The inflammation–ncRNA interface becomes most consequential when it crosses into irreversible injury. miR-181c-5p suppresses NLR family pyrin domain containing 1 (NLRP1)-dependent pyroptosis, miR-7a–KLF4 links inflammatory signaling to ferroptotic vulnerability, and astrocytic miR-10b-3p–NOX4 provides a non-cell-autonomous route to neuronal ferroptosis (49, 65, 66). These mechanisms distinguish regulation of inflammatory state from regulation of cell fate.

Patient-derived neuronal EV studies show that disease-associated neuronal vesicles can influence immune cells: neuron-associated vesicles from patients with AD carry altered miRNA profiles and provoke microglial responses (9), while miR-124-3p contributes to an IFNγ-responsive microglial program elicited by mutant-APP neuronal signals (67). These observations support a feedback route from neuronal proteotoxic stress to microglial state and back to neuronal function, but the presence of a candidate miRNA within an active EV preparation does not by itself establish that the RNA is necessary or sufficient for the vesicle-level phenotype; RNA-specific depletion or rescue is required for that attribution.

Human and multi-omics studies identify inflammatory ceRNA networks, circulating miRNAs, spatiotemporal neuron–glia signatures and disease-linked circRNAs (68–72), but their causal depth varies. Human association should therefore be treated as a distinct evidence dimension rather than as a substitute for perturbational mechanism.

### 3.4 Neurovascular, peripheral and multicellular interfaces

The neurovascular evidence is comparatively sparse, which is itself informative. IL-1β induces endothelial miRNAs that suppress low-density lipoprotein receptor-related protein 1 (LRP1)-mediated brain-to-blood Aβ transport (73), linking inflammation to a vascular clearance mechanism. At the blood–CSF interface, choroid-plexus-derived exosomal miR-146a can modify hippocampal inflammatory signaling (74). These studies identify brain interfaces as mechanistic gaps rather than peripheral footnotes.

Peripheral ncRNAs are analytically accessible but mechanistically ambiguous. In AD peripheral blood mononuclear cells (PBMCs), anti-inflammatory effects of klotho are associated with increased miR-29a (75), whereas extracellular miR-29a-5p activates mouse TLR7 and human TLR7/8 in receptor-reporter assays, with functional neuroinflammatory effects demonstrated primarily as TLR7-dependent in murine microglia and *in vivo* (11). The contrast separates three claims that are often conflated: a circulating ncRNA can mark disease, regulate systemic immunity or causally communicate with the CNS. Evidence is currently strongest for the first two.

The cellular evidence supports a distributed neuroimmune network. Neuronal stress alters astrocytic and microglial RNA programs (50); neuron-derived EVs modify microglia (9, 67); microglia regulate Aβ handling and release extracellular RNA (6, 7, 76); and vascular or barrier tissues influence clearance and inflammatory signaling (73, 74) (**Figure 1**).

**Figure 1:**
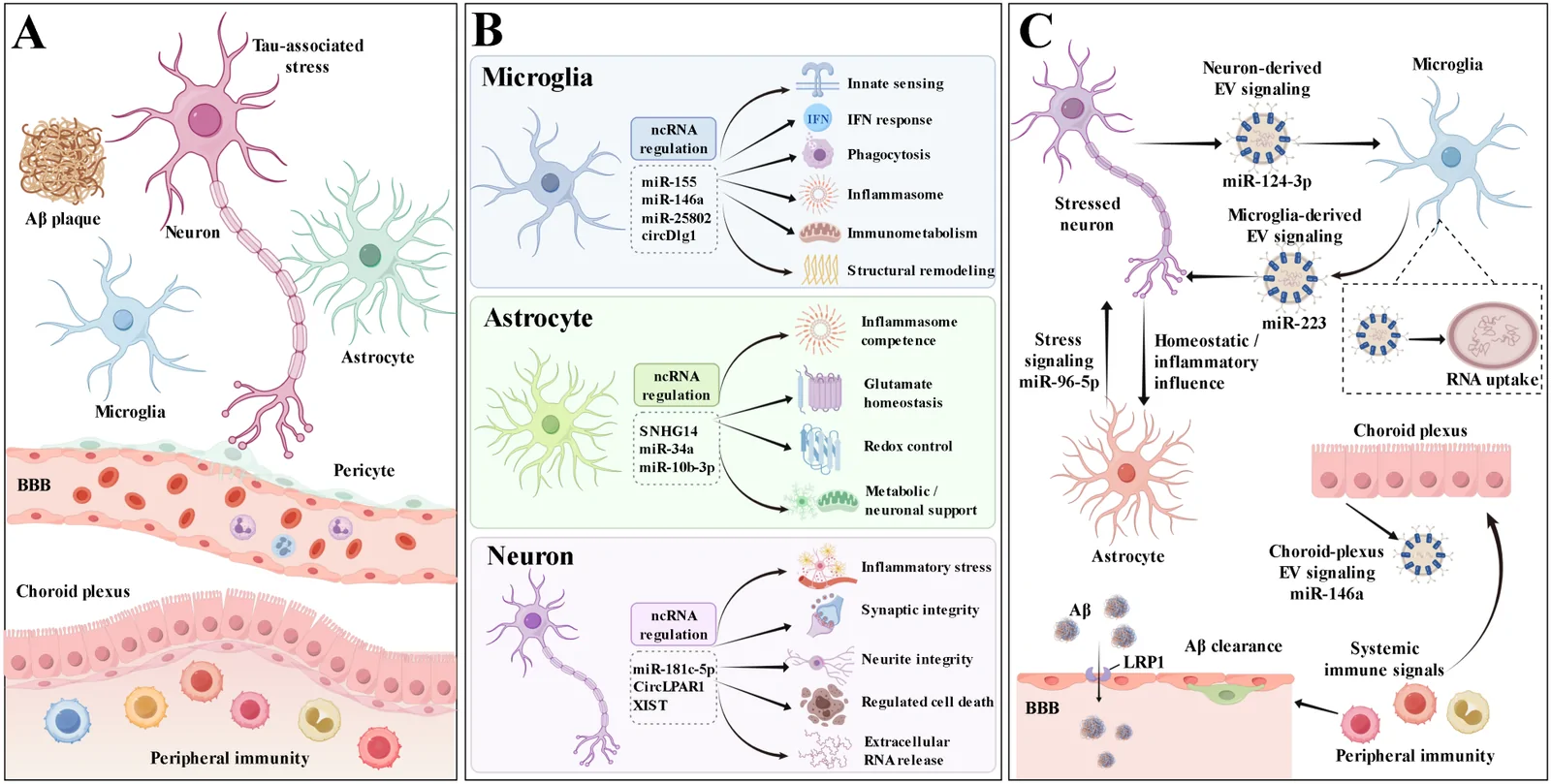
The ncRNA-regulated neuroimmune ecosystem in Alzheimer’s disease. **(A)** Multicellular organization of the AD neuroimmune environment, including microglia, astrocytes, neurons, Aβ plaques, tau-associated neuronal stress, the BBB, pericytes, choroid plexus and peripheral immunity. **(B)** Representative ncRNA-regulated programs in major neural cell types. Microglial ncRNAs regulate innate sensing, interferon responses, phagocytosis, inflammasome activity, immunometabolism and structural remodeling; astrocytic ncRNAs regulate inflammasome competence, glutamate homeostasis, redox control and metabolic/neuronal support; neuronal ncRNAs regulate inflammatory stress, synaptic and neurite integrity, regulated cell death and extracellular RNA release. Representative ncRNAs are indicated in each module. **(C)** Intercellular ncRNA communication across the AD neuroimmune ecosystem. Neuron-derived EVs, including miR-124-3p, signal to microglia, whereas microglial EVs carrying miR-223 signal to neurons. Neuron–astrocyte communication, choroid-plexus EV signaling via miR-146a, LRP1-mediated brain-to-blood Aβ transport across the BBB, peripheral immune influences and EV RNA uptake further connect parenchymal and brain-interface compartments. Representative pathways illustrate how ncRNA-regulated cell states are integrated through multicellular communication. This figure was created using Figdraw.

## 4 Molecular architectures of ncRNA regulation in Alzheimer’s neuroinflammation

Cell-state programs are implemented through a limited set of molecular architectures: miRNA-mediated repression, RNA–RNA networks, RNA–protein interactions, control of molecular stability, epigenetic regulation, and RNA modification. Their convergence on the same inflammatory outputs makes mechanism—not RNA class—the more informative organizing principle.

### 4.1 Canonical and networked RNA regulation

Canonical miRNA-mediated repression remains the dominant architecture. Comparatively well-resolved examples include miR-25802–KLF4, miR-181c-5p–NLRP1, miR-7a–KLF4 and miR-212-3p–specificity protein 1 (SP1)/beta-site amyloid precursor protein cleaving enzyme 1 (BACE1)/NLRP3, alongside multi-omics and MAPK-centered pathways (6, 21, 23, 28, 65, 66, 77). Because transcription factors, inflammasome components and kinases control broad programs, a single miRNA–target interaction can propagate across inflammation, metabolism and cell fate. Target prediction or reporter binding is therefore necessary but insufficient: the interaction must account for a meaningful fraction of the disease-relevant phenotype at endogenous concentrations.

Proposed lncRNA-centered networks include NEAT1–miR-361-3p–TRAF2, SNHG3–miR-128-3p–UBR5, SNHG14–miR-223-3p–NLRP3, MAGI2-AS3–miR-374b-5p and PRR34-AS1–miR-29c-3p (8, 14, 35, 42, 44), with additional ceRNA analyses expanding the network space (40). Their biological plausibility is strongest when RNA interaction is matched by endogenous perturbation and functional rescue, not merely reciprocal expression. A convincing ceRNA mechanism should establish co-localization, plausible stoichiometry, direct interaction and functional consequence at endogenous RNA levels; otherwise, overexpression risks creating competition that is absent in disease tissue.

### 4.2 Non-canonical RNA mechanisms

circRNA biology is broader than the early miRNA-sponge paradigm. circ_0000950 and related neuronal circRNAs fit ceRNA-like models (59, 72, 78), but circDlg1 controls PDE4B stability through ubiquitination-dependent mechanisms (7), and CircLPAR1 can destabilize growth differentiation factor 15 (GDF-15) mRNA while also participating in a miR-212-3p–ZNF217 feedback network (60, 61). Function should therefore be measured, not inferred from circularity or predicted binding sites.

RNA–protein interactions add a less explored layer of regulation. circDlg1 alters PDE4B degradation, Tincr engages the MYPT1–non-muscle myosin IIA machinery, Y-box binding protein 1 (YB-1) sorts miR-223 into microglial exosomes and CircLPAR1 alters GDF-15 mRNA stability (7, 29, 60, 76). These mechanisms extend ncRNA regulation from transcript abundance to the organization and turnover of molecular complexes.

Nuclear and epitranscriptomic mechanisms further expand this repertoire. XIST-mediated repression of neprilysin links a nuclear lncRNA program to Aβ degradation and inflammation (57), whereas METTL3-dependent m6A regulation of BDNF-AS links RNA-modification machinery to ncRNA fate and neuroinflammatory signaling (79). These mechanisms remain less mature than canonical miRNA biology but broaden the types of RNA regulation relevant to AD.

### 4.3 Emerging RNA classes and mechanistic diversity

The field remains dominated by miRNAs, but tRF(Ala-AGC-3-M8) shows that other small-RNA classes can regulate ERK/p70S6K-associated neuroinflammatory programs (22). Evidence for piRNAs and several additional classes remains too sparse for equivalent mechanistic claims, marking a discovery gap rather than a mature subfield.

Across these mechanisms, RNA class is a poor proxy for function: circRNAs can control protein stability (7), miRNAs can become extracellular innate immune ligands (11), and tRFs can converge on classical inflammatory pathways (22). Mechanism, cell type and compartment provide the more useful bridge to intercellular signaling (**Table 1**).

**Table 1:** Selected ncRNA mechanisms with comparatively strong mechanistic evidence linking neuroinflammation to Alzheimer’s disease.

ncRNA	Cell/compartment	Key mechanism	Integrated neuroimmune/AD consequence	Evidence profile/primary model context	References
miR-155	Microglia	IFNγ-associated state transitions and phagocytic programs	Amyloid handling is coupled to network-level trade-offs; complete microglial deletion lowers Aβ but causes hyperexcitability and seizures	Microglia-specific genetic perturbation in transgenic AD mouse models; strong causal evidence with context-dependent network trade-offs	(4, 5)
miR-25802	Microglia	KLF4–NF-κB regulation	Controls neuroinflammation, Aβ-related pathology and cognition in microglial/*in vivo* models; opposing protection after sEV delivery was reported only in a distinct Aβ-treated SH-SY5Y *in vitro* system	Microglial cellular and *in vivo* AD models with KLF4 rescue plus peripheral human association; direction cannot be generalized to the distinct sEV/SH-SY5Y study	(6)
circDlg1	Microglia	RNA–protein interaction controlling PDE4B ubiquitination-dependent degradation	Links inflammatory signaling, microglial function, Aβ pathology and cognition	APP/PS1 microglia and mice plus post-mortem human AD brain; comparatively strong human–mechanistic convergence	(7)
SNHG3/miR-128-3p	Microglia; neurons	lncRNA–miRNA–UBR5 regulatory network	Couples neuroinflammation and oxidative stress to cognitive impairment	AD-related cellular/*in vivo* models with human-associated evidence; mechanistic support is substantial, but ceRNA stoichiometry remains a consideration	(8)
miR-29a-5p	Extracellular; microglial sensing	Reporter activation of mouse TLR7 and human TLR7/8; downstream functional neuroinflammation primarily TLR7-dependent	Innate immune activation and neuronal injury; enhanced Aβ phagocytosis reflects uptake and does not by itself establish net Aβ clearance	TLR reporter systems, primary murine microglia, and WT/APP/PS1 mice; human TLR7/8 reporter activation, but functional neuroinflammatory effects primarily TLR7-dependent	(11)
miR-34a	Microglia	Nucleic-acid sensing and type I interferon programs	Deficiency enhances interferon-responsive states while improving selected AD outcomes	Genetic deficiency in an AD mouse model; strong causal evidence with marked state dependence	(19)
miR-22	Microglia/inflammatory injury	Inflammasome- and pyroptosis-associated regulation	Modifies neuroinflammation, neuronal injury and cognition	AD-related preclinical models plus peripheral patient association; mechanistic evidence for inflammasome/pyroptosis-related effects	(20)
miR-28a-5p	Microglia	SerpinA3N–PI3K/AKT axis identified by multi-omics	Regulates microglial inflammation and cognitive decline	Multi-omics-guided preclinical *in vivo* AD study; causal support is strong within the model, with limited direct human validation	(21)
tRF(Ala-AGC-3-M8)	Microglia; neurons	EphA7–ERK–p70S6K signaling	Reduces neuroinflammation and neuronal injury with Aβ/tau-related effects	Preclinical cellular and *in vivo* AD-related models; mechanistic evidence extends beyond canonical miRNAs, with limited human validation	(22)
miR-146a	Microglia; EV-associated compartments	Microglial state regulation and inflammatory feedback	Influences Aβ handling, cognition and inflammatory adaptation	Microglial, EV-associated and inflammatory contexts across preclinical models; effects vary with compartment and inflammatory history	(31, 82, 83)
miR-181c-5p	Microglia; neurons	Aβ-responsive microglial function and NLRP1-dependent neuronal pyroptosis	Links phagocytic competence to neuronal inflammatory death through distinct cell-specific mechanisms	Cell-specific preclinical evidence in microglial Aβ handling and neuronal NLRP1-dependent pyroptosis; concordant but pleiotropic	(32, 65)
miR-132	Patient-derived microglia	Inflammatory-state modulation	Attenuates inflammatory responses in AD patient-derived microglia	Direct perturbation in AD patient-derived iPSC microglia; human causal cellular model without whole-organism validation	(43)
SNHG14/miR-223-3p	Astrocytes	ceRNA regulation of NLRP3	Controls astrocyte-mediated neuroinflammation and neuroprotection	AD-related cellular and *in vivo* preclinical models with rescue evidence; no direct patient-derived material	(44)
miR-96-5p	Neuron–astrocyte circuit	Cathepsin B-associated neuron-derived inflammatory signaling	Regulates astrocyte reactivity and memory phenotypes	Neuron–astrocyte preclinical circuit with *in vivo* causal evidence and human-associated support	(50)
XIST	Neurons	Epigenetic repression of neprilysin	Promotes Aβ accumulation and neuroinflammatory injury	Predominantly preclinical neuronal/AD-related models; mechanistic nuclear lncRNA evidence with limited direct human validation	(57)
CircLPAR1	Neurons	GDF-15 mRNA destabilization and miR-212-3p–ZNF217 feedback regulation	Promotes oxidative stress, inflammation and neuronal injury	Predominantly preclinical neuronal/AD-related models; multiple mechanisms support pleiotropy, but direct human validation remains limited	(60, 61)
miR-7a	Neuronal/vascular-associated contexts	KLF4-dependent inflammatory and ferroptotic regulation	Couples neuroinflammation to ferroptotic vulnerability	Preclinical *in vivo* AD model with KLF4-dependent inflammatory/ferroptotic regulation; limited direct human support	(66)

Evidence profiles report causal strength, mechanistic resolution, human relevance, primary experimental context and context dependence as separate dimensions; operational definitions are provided in **Box 1**.

Box 1 **Evaluating evidence for ncRNA-mediated neuroinflammation**

Causality. We classify evidence as causal only when selective gain- or loss-of-function changes a disease-relevant phenotype; confidence increases with cell-specific perturbation, disease-relevant *in vivo* validation and reproducibility across models.

Mechanistic resolution. We consider a mechanism comparatively well resolved when direct target or molecular-interaction evidence is linked to the phenotype by downstream mediation, rescue or orthogonal biochemical validation; target prediction, reciprocal expression or reporter binding alone is insufficient.

Human relevance. Human evidence is specified by source—post-mortem brain, CSF, plasma/serum, PBMCs, patient-derived EVs or disease-relevant patient-derived cells—because these materials provide different anatomical and mechanistic information; none substitutes for causal evidence.

Translational maturity. Therapeutic maturity increases when direct RNA manipulation is coupled to a defined modality, CNS and cellular delivery, target engagement, pharmacodynamic monitoring and, where available, intervention-associated human data. Treatment-responsive RNA changes are considered pharmacodynamic associations unless RNA mediation is directly demonstrated.

These dimensions address different questions and should not be collapsed into a single hierarchy of study quality. Accordingly, **Table 1** uses ‘comparatively strong mechanistic evidence’ only when the relevant dimensions are stated explicitly rather than as a global high-confidence label.

## 5 Extracellular ncRNAs and intercellular neuroimmune communication

Extracellular RNA adds a second dimension to ncRNA biology: regulatory information can be exported, transported and reinterpreted by another cell. Throughout this review, extracellular vesicle (EV) is used as the umbrella term; small EV (sEV) or exosome is retained only when the cited study provides the relevant operational characterization or when explicitly reporting the authors’ original terminology. The strongest studies resolve not only cargo, but a chain from donor-cell state through RNA selection and carrier to recipient-cell mechanism and phenotype.

### 5.1 EV sorting and donor–recipient communication

EV cargo can be selectively regulated rather than representing a diluted copy of the donor transcriptome. YB-1 promotes loading of miR-223 into microglial vesicles reported as exosomes in the original study, and perturbing this sorting step changes neuronal injury and cognition (76). Cargo selection is therefore part of both EV mechanism and therapeutic engineering.

Microglia can export protective or pathological RNA signals. miR-223-containing vesicles reported as exosomes in the original study provide a protective example (76), whereas miR-711-containing EVs and particulate-exposure-associated miR-34a-5p vesicles illustrate context-dependent effects in rmTBI and PM2.5-related models (80, 81). The mechanism may be informative even when disease-model relevance limits direct inference to sporadic AD.

Neuron-derived RNA provides a route by which proteotoxic stress can reprogram microglia. Patient-derived neuron-associated EVs alter human microglial responses (9), and miR-124-3p contributes to an IFNγ-responsive microglial signature elicited by mutant amyloid precursor protein (APP) neuronal signaling (67). Together, these findings connect neuronal stress with extracellular RNA signaling, microglial state changes and subsequent alterations in the neuronal environment.

MiR-146a illustrates why EV function is context-dependent. Exosomal miR-146a participates in TLR tolerance, whereas miR-146a dysregulation can accompany energetic dysfunction during neuroinflammation (82, 83); miR-21-5p-associated neuron–glia dysregulation adds a further example of source- and recipient-dependent signaling (84). EV-associated miRNAs should therefore be interpreted as components of dynamic feedback programs, not assigned fixed beneficial or harmful labels.

Choroid-plexus-derived exosomal miR-146a links environmental enrichment to reduced hippocampal inflammatory signaling and preserved cognition (74). Although based on a limited evidence set, this finding expands ncRNA communication beyond the parenchymal neuron–glia unit and identifies barrier tissues as potential signaling hubs.

### 5.2 Extracellular RNA as an innate immune signal

Extracellular miR-29a-5p directly activates mouse TLR7 and human TLR7/8 in receptor-reporter systems (11), a mechanism fundamentally different from intracellular RISC-mediated repression. In the same study, cytokine responses, microglia-associated neuronal injury and the short-term *in vivo* neuroinflammatory phenotype were functionally demonstrated primarily as TLR7-dependent. Patient-derived microglial EVs carrying inflammatory miRNAs reinforce the plausibility of RNA-driven innate activation in human AD-associated material (10), but cargo detection alone does not identify the causal RNA species. Compartment can therefore switch the molecular identity—and potentially the direction—of an ncRNA effect.

### 5.3 From extracellular cargo to functional communication

Altered RNA abundance in an EV is not evidence of communication by itself, and a biological effect of an EV preparation does not establish that a measured RNA cargo mediates that effect. Stronger studies define donor-cell origin, reproducible enrichment, recipient delivery and RNA-specific loss or rescue of function. YB-1/miR-223, patient neuronal EVs and patient microglial EVs each satisfy different parts of this causal chain (9, 10, 76); where RNA-specific necessity or sufficiency has not been tested, we describe the cargo as associated with, rather than proven to mediate, the vesicle phenotype.

Extracellular RNA exists in multiple vesicular and non-vesicular complexes. Carrier state shapes stability, biodistribution, receptor access and intracellular trafficking; for endosomal TLR signaling, it may determine whether an RNA acts as an immune ligand or reaches cytoplasmic silencing machinery (11). Precise terminology is therefore mechanistic, not merely methodological.

Extracellular RNA allows dysregulation to propagate across cells: stressed neurons alter microglia (9), microglia export protective or pathogenic RNA cargo (10, 76), and extracellular RNA can directly engage innate receptors (11). This distributed signaling layer closes the loop between cell-state regulation and AD pathology, where ncRNA-dependent immune responses feed back onto proteostasis, metabolism and neuronal function (**Figure 2**).

**Figure 2:**
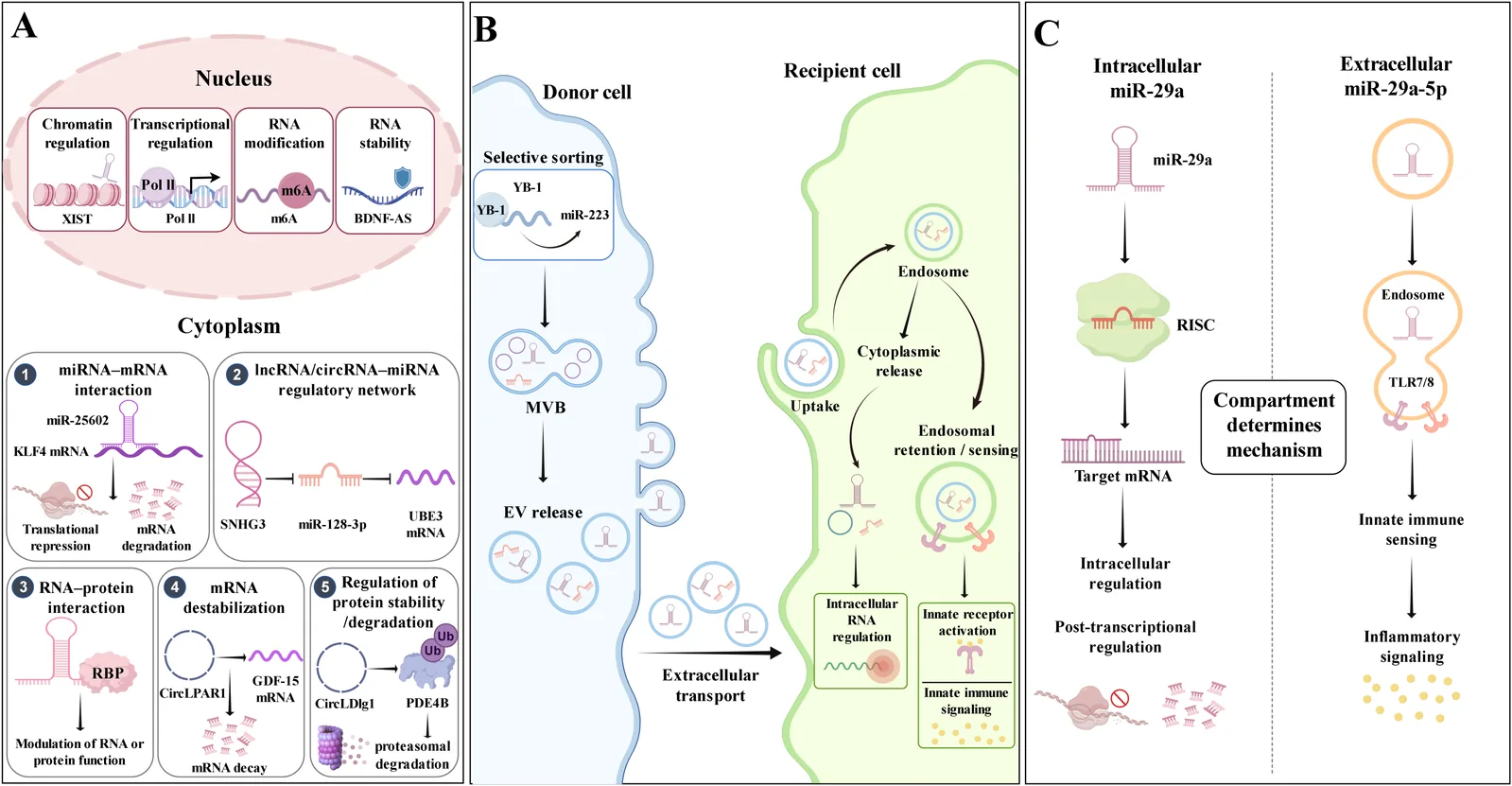
Molecular and compartmental mechanisms of ncRNA regulation. **(A)** Intracellular ncRNA mechanisms include chromatin and transcriptional regulation, RNA modification and stability, miRNA–mRNA repression, lncRNA/circRNA–miRNA regulatory networks, RNA–protein interactions, mRNA destabilization, and regulation of protein stability. Representative examples include XIST, BDNF-AS/m6A, miR-25802–KLF4, SNHG3–miR-128-3p–UBR5, CircLPAR1–GDF-15 and circDlg1–PDE4B. **(B)** Extracellular RNA communication involves selective ncRNA sorting, EV biogenesis and release, extracellular transport, recipient-cell uptake and endosomal trafficking. YB-1-mediated sorting of miR-223 illustrates selective EV cargo loading. Following uptake, RNA cargo may undergo productive cytoplasmic release and intracellular regulation or remain endosomally retained and engage innate immune sensing. **(C)** Compartment can switch ncRNA mechanism. Intracellular miR-29a acts through RISC-mediated post-transcriptional regulation, whereas extracellular miR-29a-5p can enter endosomes, activate TLR7/8 and promote innate inflammatory signaling. Together, these mechanisms illustrate that ncRNA function is determined by both molecular architecture and cellular compartment. This figure was created using Figdraw.

## 6 ncRNA-controlled neuroinflammation as a modifier of Alzheimer’s disease pathology

ncRNA-regulated inflammation matters to AD only insofar as it reshapes disease-defining pathology. Across the evidence base, inflammatory RNA programs intersect with Aβ production and clearance, tau pathology, synaptic dysfunction, metabolic stress and regulated cell death. The recurring architecture is reciprocal rather than linear: proteotoxic stress changes ncRNAs, ncRNAs reprogram immunity, and the altered immune state feeds back onto pathology.

### 6.1 Amyloid production, clearance, and inflammatory feedback

ncRNAs regulate Aβ homeostasis at distinct levels. miR-212-3p links SP1/BACE1 to NLRP3–caspase-1 signaling (77); XIST affects neprilysin-dependent degradation (57); and endothelial miRNAs suppress LRP1-mediated brain-to-blood transport (73). Microglial pathways involving miR-25802, miR-146a, miR-181c-5p and circDlg1 further shape uptake and processing (6, 7, 31, 32). ‘Reduced Aβ’ therefore conflates biologically distinct processes—production, internalization, lysosomal degradation and vascular export.

Aβ can alter ncRNA expression across microglia, astrocytes and neurons; the resulting RNA programs modify inflammatory and stress pathways that feed back onto amyloid processing or clearance. hsa_circ_0049472 and miR-204-3p/FBXL7 provide additional examples of this loop architecture (85, 86). These interactions are self-reinforcing and cannot be reduced to isolated ncRNA–target pairs.

### 6.2 Tau, synaptic dysfunction, and network consequences

Tau-centered evidence is substantially less mature than the Aβ literature. miR-146a-associated energetic dysfunction, tRF(Ala-AGC-3-M8), miR-485-3p antisense therapy and nilotinib-associated CSF miRNA changes connect ncRNA-controlled inflammation or metabolism to tau-related outcomes (12, 22, 83, 87). Direct control of tau propagation or tau-responsive microglial states, however, remains insufficiently resolved for claims equivalent to those in Aβ biology.

ncRNA-controlled inflammation can influence cognition independently of plaque burden. miR-98-5p regulation of α7 nicotinic acetylcholine receptors couples post-transcriptional control to neuroinflammation and cognition (88), while inflammatory miR-146a/miR-155 signatures associate with mild cognitive impairment (89). Together with the miR-155 deletion phenotype (5) and astrocytic miR-181a–GLT-1 regulation (48), these data make synaptic and network function essential endpoints rather than corollaries of Aβ reduction.

### 6.3 Metabolic stress and regulated cell death

Inflammation and metabolic dysfunction also form a reciprocal loop. miR-146a dysregulation, SNHG3-associated pathways, CircLPAR1, miR-7a–KLF4 and astrocytic miR-10b-3p–NOX4 connect ncRNA regulation to energetic stress, oxidative injury and cell-death susceptibility (8, 49, 60, 66, 83). Metabolic competence is therefore part of neuroimmune state, not simply a downstream consequence of inflammation. Magnolol-associated miR-200c upregulation in an insulin-resistant AD model provides pharmacological support for coordinated metabolic, inflammatory and cognitive responses (90), although the RNA change cannot be assumed to mediate the full drug effect.

miR-181c-5p–NLRP1, miR-22, SNHG14–miR-223-3p–NLRP3, miR-224-5p–NLRP3 and miR-7a–KLF4 converge on pyroptotic or ferroptotic programs (20, 44, 46, 65, 66), with GATA4–SNHG1–miR-361-3p providing an additional link to neuroinflammation and cognition (91). Such claims should require pathway-specific rescue because marker changes alone do not establish a regulated mode of cell death.

### 6.4 Pathological coupling and systems-level outcomes

miR-155 provides the clearest warning against single-endpoint efficacy. Partial inhibition can reduce neuroinflammation and cognitive impairment (92), whereas complete microglial deletion lowers amyloid pathology but causes severe hyperexcitability and seizures (5). Genetic deletion, partial inhibition and cell-selective modulation are not interchangeable; therapeutic assessment must span proteopathy, neuroimmune state, network function and cognition.

We therefore propose a conceptual, non-quantitative feedback-gain framework for integrating these observations (**Figure 3**). Here, ‘gain’ is a heuristic description of how strongly pathological domains reinforce one another, not an experimentally measured parameter. Experimentally supported relationships include ncRNA effects on inflammatory signaling, Aβ production or handling, metabolic stress and neuronal/network phenotypes; proposed cross-domain feedbacks are presented as hypotheses generated by synthesis of the available literature. This systems-level view explains both the therapeutic appeal and liability of ncRNAs: the tractable target is a feedback node whose modulation preserves compensatory function rather than maximally suppressing a single pathway.

**Figure 3:**
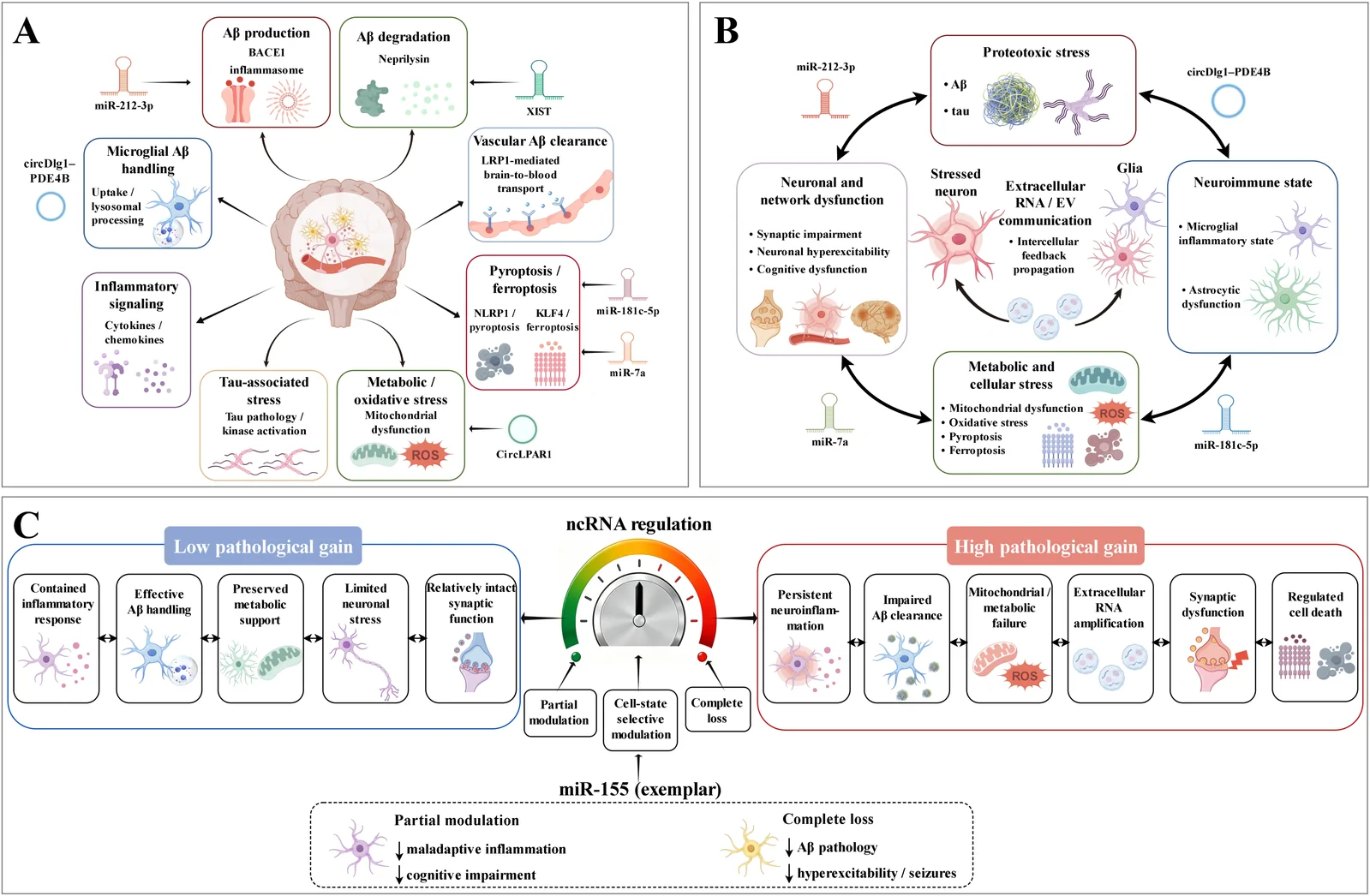
Conceptual, non-quantitative framework for ncRNA-regulated pathological feedback in Alzheimer’s disease. **(A)** Representative ncRNAs regulate multiple entry points into AD pathology, including Aβ production and degradation, microglial Aβ handling, LRP1-mediated vascular Aβ export, inflammatory signaling, tau-associated stress, metabolic/oxidative stress, and pyroptosis or ferroptosis. Illustrative regulators include miR-212-3p, XIST, circDlg1–PDE4B, CircLPAR1, miR-181c-5p and miR-7a. **(B)** Experimentally supported relationships across these domains motivate a literature-synthesis model in which proteotoxic stress, neuroimmune state, metabolic/cellular stress and neuronal/network dysfunction can reinforce one another. Extracellular RNA and EV communication provides an additional proposed route for intercellular propagation. **(C)** ‘Pathological gain’ is used here as a heuristic, not an experimentally measured quantity. Low-feedback-gain states represent contained inflammation, effective Aβ handling, preserved metabolic support and neuronal function, whereas high-feedback-gain states represent persistent neuroinflammation, impaired Aβ handling/disposal, metabolic failure, extracellular RNA amplification, synaptic dysfunction and regulated cell death. miR-155 illustrates why the degree and cellular context of modulation matter: partial inhibition can reduce maladaptive inflammation, whereas complete microglial loss can reduce amyloid pathology while promoting hyperexcitability and seizures. Solid mechanistic links reflect cited perturbational evidence; broader cross-domain coupling is conceptual and intended to generate testable hypotheses. This figure was created using Figdraw.

## 7 From association to causality: interpreting ncRNA evidence

The field now contains many AD-associated RNA signatures but far fewer established causal regulators. Causality, molecular closure, human relevance and translational maturity overlap only partly; differential expression, target validation, *in vivo* phenotype and patient association therefore need to be interpreted as distinct evidence layers.

### 7.1 From expression to causal mechanism

An ncRNA that differs between AD and control tissue may drive pathology, compensate for it or simply report altered cell composition. A stronger evidential sequence moves from cell- and compartment-resolved association to selective perturbation, functional change, disease-relevant *in vivo* phenotype, molecular rescue and human mechanistic convergence (5–7, 9, 10, 14, 43, 44, 65).

Causality and mechanistic closure are related but non-equivalent. miR-155 studies establish strong biological causality without reducing the phenotype to one direct target (4, 5), whereas more restricted target or ceRNA studies can achieve high molecular closure. miR-25802–KLF4–NF-κB and circDlg1–PDE4B exemplify convergence of the two (6, 7). A binding interaction is therefore not, by itself, a disease mechanism.

Target rescue is most informative when restoring the downstream node reverses a disease-relevant phenotype, as for miR-25802–KLF4 and miR-181c-5p–NLRP1 (6, 65). Supraphysiological overexpression, however, can manufacture apparent rescue; physiological RNA levels and functional endpoints remain essential.

ceRNA studies illustrate the distinction between molecular interaction and disease causality: NEAT1-, SNHG14- and SNHG3-centered networks provide comparatively strong examples (8, 14, 44), whereas circDlg1 demonstrates that circRNA function can instead depend on protein stability (7).

### 7.2 Human relevance and context dependence

Patient brain, CSF, blood or EV evidence increases disease relevance without proving causality. Human iPSC microglia (43) and patient-derived EV functional studies (9, 10) are especially informative because they add perturbation or recipient-cell testing to a human disease-associated context.

Five cases define the main axes of context dependence. miR-155 exposes trade-offs between amyloid handling, immune state and network stability (4, 5). miR-146a can restrain inflammatory signaling, participate in EV-associated tolerance and accompany energetic dysfunction (31, 82, 83). miR-34a changes direction across cell states (19). For miR-29a, intracellular immune regulation contrasts with extracellular receptor activity: miR-29a-5p activates human TLR7/8 in reporter systems, whereas the neuroinflammatory phenotype in murine microglia and *in vivo* was demonstrated predominantly as TLR7-dependent (11, 75). For miR-25802, Zhao et al. showed in microglial and *in vivo* AD models that overexpression aggravated neuroinflammation, Aβ deposition and cognitive impairment, whereas inhibition was beneficial and KLF4 manipulation provided downstream rescue (6). By contrast, Çelik et al. reported protection after loading miR-25802 into milk-derived sEVs and applying them to Aβ-exposed SH-SY5Y human neuroblastoma cells, an *in vitro* neuronal model (93). Differences in recipient cell, carrier, delivered dose and disease context are plausible explanations for the opposing directions, but they have not been tested head-to-head and therefore remain hypotheses rather than a demonstrated compartment switch.

A mechanistic ncRNA claim should define the relevant RNA, source and recipient cells, compartment, disease stage, dose and functional endpoint. Whole-brain measurements, immortalized cell lines and single-stage perturbations obscure these variables. Model relevance must also be explicit: ultrafine-particle, PM2.5 and rmTBI EV studies identify useful mechanisms (36, 80, 81) but should not be weighted as direct evidence for sporadic AD (**Box 2**).

Box 2 **Common experimental and interpretive pitfalls**

Cell states. M1/M2 terminology should not be treated as fixed microglial or astrocytic identities. When retained to describe an original study, the label is treated as study-reported shorthand and interpreted through the measured functional outputs.

Differential expression. Disease-associated abundance identifies candidates but does not establish a causal mechanism.

ceRNA mechanisms. Binding and reciprocal expression require co-localization, plausible endogenous stoichiometry and functional rescue before competition can be considered physiologically meaningful.

Peripheral samples. Blood or PBMC signals support systemic biology or biomarker associations unless a CNS source, route and recipient-cell mechanism are established.

Human cell lines. Established human cell lines are not equivalent to patient tissue, patient-derived extracellular vesicles or disease-relevant patient-derived cells.

EV cargo. Detection of an RNA inside EVs does not prove functional transfer, and an EV-level phenotype does not prove that a measured RNA cargo is causal; donor origin, recipient uptake and RNA-specific loss or rescue should be demonstrated.

EV terminology. EV is used as an umbrella term; ‘small EV’ and ‘exosome’ are reserved for studies with the relevant operational characterization or are explicitly identified as the terminology used by the original authors.

Compartment. Intracellular and extracellular forms of the same RNA can engage different mechanisms, including canonical target repression and innate RNA sensing.

Amyloid handling. Aβ uptake should not be equated with effective lysosomal degradation or net clearance.

Model relevance. Environmental or injury-induced AD-like models can identify modifying mechanisms but should not be weighted as direct evidence for sporadic AD.

Therapeutic delivery. BBB passage, tissue accumulation, cellular uptake, endosomal escape/productive intracellular exposure and molecular target engagement are distinct steps; success at one should not be used as a surrogate for the next.

Innate sensing. Therapeutic RNA can itself activate nucleic-acid sensors; sequence-, chemistry-, structure-, purity- and trafficking-dependent immunogenicity is part of mechanism as well as safety.

### 7.3 Principles for evidence prioritization

Evidence prioritization is strengthened by considering four dimensions separately (**Box 1**): causal perturbation of a disease-relevant phenotype, direct molecular interaction and downstream mediation, support from explicitly identified human material, and progress toward therapeutic manipulation or delivery. We use ‘causal perturbation’ only when selective gain- or loss-of-function changes a disease-relevant phenotype; ‘mechanistic resolution’ requires direct target or molecular-interaction evidence linked to the phenotype by mediation, rescue or orthogonal validation; ‘human relevance’ specifies whether evidence derives from brain tissue, CSF, plasma/serum, PBMCs, patient-derived EVs or patient-derived cells; and ‘translational maturity’ refers to direct RNA manipulation, delivery, target engagement or pharmacodynamic monitoring. These dimensions should not be collapsed into a single quality ladder (6, 7, 12, 43), and descriptors such as ‘comparatively strong’ refer only to convergence across specified dimensions rather than to a universal grade of study quality.

## 8 Human evidence and biomarker potential

Human ncRNA evidence is most informative when disease-associated changes can be assigned to a cell state, mechanism, disease stage or treatment response. Human association, mechanistic convergence and intervention responsiveness should therefore be considered separately.

### 8.1 Brain, CSF, and peripheral biofluids

Post-mortem brain provides anatomical relevance but predominantly late-stage information. circDlg1 is particularly informative because human disease-associated changes converge with a resolved microglial RNA–protein mechanism (7); AOAH/miR-450b-5p, RN7SL1 regional profiling and human ceRNA analyses provide supporting evidence at different levels of maturity (17, 30, 69). Human brain data are most persuasive when paired with cell-specific perturbation rather than used as stand-alone causality.

CSF is CNS-proximal but heterogeneous in cellular origin. miR-485-3p combines brain, CSF and plasma detection with direct antisense intervention (12), whereas patient-derived EV and miR-146a-associated studies illustrate how CSF-proximal RNA may report inflammatory or metabolic state (10, 15, 83). Its strongest niche may therefore be state or pharmacodynamic monitoring rather than diagnostic competition with established Aβ and p-tau assays.

Blood enables repeated sampling but sacrifices anatomical specificity. Peripheral evidence can reinforce mechanisms involving miR-25802, SNHG3, MAGI2-AS3 and inflammatory miRNAs (6, 8, 35, 89), yet PBMC biology cannot be assumed to represent central nervous system (CNS) inflammation. The intracellular-versus-extracellular miR-29a contrast makes this limitation explicit (11, 75).

### 8.2 Patient-derived EVs and cellular systems

Patient-derived EVs move human evidence closer to mechanism because disease-associated material can be tested directly on recipient cells. Neuron-associated and microglial EVs from patients with AD alter immune responses (9, 10). The strongest designs combine cell-of-origin validation with quantitative cargo measurements and RNA-specific depletion or rescue.

Patient-derived iPSC microglia permit direct ncRNA perturbation in a human genetic background; miR-132 is a leading example (43). Their main limitation is incomplete recapitulation of biological aging, making integration with aged tissue and multicellular human models important for mechanism validation.

### 8.3 Intervention, biomarkers, and longitudinal resolution

Intervention studies add temporal information unavailable from cross-sectional profiling. Nilotinib alters CSF miRNAs alongside inflammatory and Aβ/tau-related biomarkers, exercise modifies miR-129-5p with cognitive and inflammatory outcomes, and simvastatin changes miR-106b in clinical and experimental settings (87, 94, 95). These data establish treatment responsiveness, not RNA mediation.

Current human evidence remains dominated by cross-sectional and compartment-averaged measurements, limiting assignment of ncRNA signals to specific cell states and disease stages (96, 97) (**Table 2**).

**Table 2:** Human evidence for ncRNAs in Alzheimer’s neuroinflammation.

ncRNA/signature	Human material	Principal finding	Mechanistic status	Potential use/major limitation	References
circDlg1	Post-mortem AD brain	Disease-associated change converges with a resolved microglial PDE4B stability mechanism	Human–mechanistic convergence	Mechanistic validation; late-stage tissue limits temporal inference	(7)
miR-132	AD patient-derived iPSC microglia	Direct perturbation attenuates inflammatory responses	Human causal cellular model	Useful for target validation; reprogramming incompletely preserves aging biology	(43)
miR-25802	Peripheral blood/PBMC-associated evidence	Peripheral human association supports convergence with a KLF4–NF-κB mechanism defined in microglial and *in vivo* preclinical models	Human association plus strong experimental mechanism	Potential stratification marker; peripheral source does not establish CNS origin	(6)
miR-22	Peripheral patient samples	Peripheral patient association converges with inflammasome/pyroptosis-related effects defined in preclinical models	Human association plus mechanistic support	Potential inflammatory-state marker; CNS specificity remains uncertain	(20)
let-7e	Patient neuron-derived EVs	AD-associated EV cargo modifies inflammatory responses in human microglia	Functional patient-derived EV evidence	Candidate intercellular-state biomarker; quantitative cargo sufficiency remains unresolved	(9)
Multiple EV miRNAs	Patient-derived microglial EVs/CSF-associated material	Disease-derived EV preparations alter recipient-cell neuroinflammatory responses; individual RNA-cargo causality requires RNA-specific depletion/rescue	Functional human EV evidence	Strong bridge from association to function; cell-of-origin and cargo attribution remain important	(10)
miR-485-3p	Brain, CSF and plasma	Human detection converges with direct antisense efficacy in AD models	Human-supported therapeutic candidate	Potential theranostic marker; mechanistic target network is incompletely resolved	(12)
miR-450b-5p/AOAH	Post-mortem AD brain	Links impaired LPS detoxification to pro-inflammatory signaling	Human tissue-associated mechanism	Supports innate-immune relevance; late-stage tissue limits causal timing	(17)
RN7SL1	Human hippocampal–entorhinal profiling	Regional ncRNA differences are linked to neuroimmune modulation	Regional human profiling	Supports anatomical stratification; causal role remains limited	(30)
CSF miRNA profile	Nilotinib intervention samples	CSF miRNAs change alongside inflammatory, vascular and Aβ/tau-related biomarkers	Human intervention/pharmacodynamic evidence	Potential treatment-response marker; RNA mediation is unproven	(87)
miR-129-5p	Exercise intervention samples	Treatment-responsive RNA change accompanies cognitive and inflammatory improvement	Human intervention-associated evidence	Potential pharmacodynamic marker; exercise has multiple upstream effects	(94)
miR-106b	Simvastatin clinical and experimental samples	Treatment-associated change accompanies memory and inflammatory outcomes	Human intervention-associated evidence	Potential pharmacodynamic marker; direct RNA mediation remains uncertain	(95)

## 9 Therapeutic translation of ncRNA neuroimmunology

ncRNAs are therapeutically attractive because they can reshape several components of an AD network at once, but the same pleiotropy creates risk. Biological target validation and delivery feasibility are therefore evaluated as separate translational axes: confidence that an ncRNA causally controls a disease-relevant state does not establish that it can be delivered safely and productively to the required CNS cell population, and an advanced carrier does not rescue weak target biology.

### 9.1 Target selection and RNA-directed modalities

Expression direction alone is insufficient for target selection because disease-associated increases can be compensatory and decreases can reflect cell loss or state transitions. Causal mechanism, human relevance, the intended cell-state effect, dose and disease stage are more informative criteria. General ncRNA therapeutics already face specificity, delivery and tolerability constraints (98); AD adds multicellular and stage-dependent neuroimmune biology.

Antisense inhibition of miR-485-3p is one of the clearest direct-RNA proofs of concept because human association converges with multi-domain preclinical efficacy (12). Yet miRNA inhibition is network tuning, not molecular deletion: the miR-155 literature shows that complete loss can differ qualitatively from partial pharmacological inhibition (5).

Pathogenic lncRNAs can, in principle, be targeted with ASOs, gapmers, siRNAs or interaction-blocking oligonucleotides, but modality should follow mechanism. A nuclear chromatin-regulating lncRNA and a cytoplasmic ceRNA are not equivalent drug targets. SNHG3, XIST, NEAT1 and SNHG14 provide proof of principle, although these pathways remain predominantly preclinical (8, 14, 44, 57).

Endogenous circRNAs such as circDlg1 and CircLPAR1 are potential targets when they drive pathology (7, 60). More transformatively, engineered circular RNA aptamers show that circRNA can itself serve as a therapeutic material (13), shifting the field from manipulating endogenous transcripts toward designing RNA architectures that reprogram pathological signaling.

### 9.2 Delivery platforms and productive intracellular exposure

Mannose-coated lipid nanoparticles targeting microglial miR-17 improve pathology and behavior in experimental AD (99), providing an AD-specific example of a broader CNS delivery principle: crossing the BBB is not equivalent to achieving cell-type or intracellular precision (100). Delivery feasibility must be decomposed into CNS exposure/BBB penetration, cell-specific targeting, cellular uptake, endosomal escape or other productive intracellular release, molecular target engagement, and acceptable off-target distribution. Evidence at one step should not be used as a surrogate for success at the next.

EV-based therapy spans increasingly diverse cargo and platforms. Hypoxia-preconditioned stem-cell vesicles reported as exosomes in the original study carry circ-Epc1 and improve inflammatory and cognitive phenotypes through a circ-Epc1–miR-770-3p–TREM2 axis (101). Mesenchymal stem cell (MSC)-derived EVs suppress NLRP3/GSDMD-mediated neuroinflammation (102); human umbilical-cord MSC vesicles reported as exosomes deliver lncRNA-9969-associated multi-target protection (103); and miR-22-, miR-137-5p-, miR-124-3p- and miR-34-containing vesicles illustrate alternative strategies (104–107). circ-Epc1/TREM2 and YB-1/miR-223 provide mechanistic links between cargo biology and outcome (76, 101). The promise is clear, but so are the product-development problems: heterogeneous cargo, uncertain dose metrics, variable EV characterization and difficulty attributing efficacy to one RNA without cargo-specific loss-of-function or rescue.

Engineered microglial EV delivery reported as exosome-mediated miR-124-3p delivery, together with MSC EVs overexpressing miR-146a, illustrates the move from native biological products toward more controlled RNA carriers (108, 109). A credible EV therapeutic will require defined cargo, reproducible loading, validated CNS distribution and target-cell specificity.

High tissue uptake is not synonymous with productive delivery. Nanoparticles and EVs can remain sequestered in endosomal or lysosomal compartments; functional efficacy therefore requires endosomal escape or otherwise productive intracellular exposure and demonstrable target engagement, not fluorescence-based uptake alone (99, 100, 108). This distinction is especially important in microglia, whose endolysosomal and nucleic-acid-sensing systems are highly active. Off-target transcript-network effects and activation of innate RNA sensors should be evaluated alongside pharmacokinetics rather than deferred to late-stage safety testing.

### 9.3 Safety, dose, stage, and pharmacodynamic monitoring

Extracellular miR-29a-5p provides a direct warning for therapeutic design: the RNA activates human TLR7/8 in reporter systems, while the experimentally demonstrated neuroinflammatory phenotype was primarily TLR7-dependent (11). Together with inflammatory effects of patient-derived EV preparations (10), this finding highlights that RNA sequence, chemistry, structure, purity, carrier and intracellular trafficking can determine whether a therapeutic molecule reaches its intended target or instead triggers innate sensing (98). Immunogenicity is therefore part of mechanism, not merely a late toxicology issue.

ncRNA therapeutic windows will depend on both disease stage and degree of modulation. miR-155 highlights the difference between complete and partial inhibition (5). Human studies of nilotinib, exercise and simvastatin show how ncRNAs can serve as pharmacodynamic readouts (87, 94, 95), whereas miR-485-3p illustrates the value of demanding efficacy across several disease domains (12).

Translational prioritization should distinguish mechanism-rich targets awaiting human validation, human-supported mechanisms awaiting a drug modality, direct RNA candidates requiring pharmacokinetic and safety development, and delivery platforms requiring rigorous target biology. RNA-medicine development increasingly integrates modality, manufacturing and clinical strategy early rather than appending them after target discovery (110) (**Table 3**).

**Table 3:** Translational landscape of ncRNA-directed interventions in Alzheimer’s neuroinflammation.

Target/cargo	Intervention	Delivery/intended target	Evidence stage	Principal outcome/key barrier	References
miR-485-3p	Antisense inhibition	Direct oligonucleotide; CNS target distribution not fully resolved	Human-supported preclinical therapeutic proof of concept	Improves Aβ, tau, neuroinflammation and cognition; cell specificity and target-network definition remain priorities	(12)
SNHG3	lncRNA knockdown	AAV-mediated silencing *in vivo*	Preclinical causal intervention	Reduces neuroinflammation and oxidative stress; long-term vector exposure and lncRNA pleiotropy require attention	(8)
miR-17	Inhibitor delivery	Mannose-coated lipid nanoparticles targeting microglia	Advanced preclinical delivery study	Improves pathology and behavior; systemic biodistribution and microglial specificity require validation	(99)
Engineered circular RNA aptamers	Synthetic RNA therapeutic	Stable engineered circular RNA	Advanced preclinical RNA platform; corrected article/source data reviewed	Corrected data continue to support amelioration of neuroinflammatory and AD-related phenotypes in mouse models; effect magnitude should be interpreted from the corrected figures/source data. Manufacturing purity, persistence and innate immunogenicity remain barriers	(13)
circ-Epc1	RNA cargo replacement	Hypoxia-preconditioned adipose-derived stem-cell EVs (reported as exosomes); TREM2-linked microglial target	Preclinical EV therapy	Improves cognition and inflammatory phenotype; RNA-specific cargo attribution, EV characterization and standardization remain limiting	(101)
miR-223-3p	EV-mediated delivery	Mesenchymal stem-cell EVs	Preclinical EV therapy	Suppresses NLRP3/GSDMD-associated neuroinflammation; cargo dose and EV heterogeneity require definition	(102)
lncRNA-9969	EV-mediated delivery	Human umbilical-cord mesenchymal stem-cell EVs (reported as exosomes)	Preclinical multi-target EV therapy	Links autophagy, lipid metabolism and microglial modulation; complex cargo complicates RNA-specific mechanism attribution	(103)
miR-22	EV-mediated delivery	Adipose-derived mesenchymal stem-cell EVs (reported as exosomes in the original study)	Preclinical EV therapy	Improves neurological and inflammatory outcomes; target-cell distribution and RNA-specific necessity/sufficiency require clarification	(104)
miR-124-3p	Engineered EV delivery	Microglial EV-based combinational therapy (reported as exosomal delivery in the original study)	Preclinical engineered EV platform	Demonstrates targeted RNA-cargo design; scalable production, reproducible CNS targeting and productive intracellular delivery remain unresolved	(108)
miR-124-3p	RNA-enriched EV treatment	RNA-enriched EV treatment tested in a neuron–glia microfluidic triculture model (reported as exosomes in the original study)	Advanced *in vitro* translational model	Supports multicellular therapeutic effects *in vitro*; RNA-specific contribution, *in vivo* pharmacokinetics and safety remain to be established	(106)

## 10 Precision ncRNA neuroimmunology: research priorities

Precision ncRNA neuroimmunology requires identifying which RNAs control disease-relevant neuroimmune states in specific cells, anatomical contexts, disease stages and patient groups rather than expanding catalogs of AD-associated ncRNAs.

### 10.1 Cell-state, spatial, and temporal resolution

Multimodal human AD atlases and ex vivo microglial datasets now define disease-relevant cell states at unprecedented resolution (96, 97). Priority should shift from differential expression to demonstrating that specific ncRNAs control entry into or exit from defined interferon-responsive, plaque-associated, phagocytic or metabolically dysfunctional states (4, 6).

Plaque proximity and local chromatin state shape microglial responses (111). ncRNA studies should therefore integrate spatial transcriptomics, RNA *in situ* methods, plaque-proximity cell isolation and spatial proteomics. This is particularly important for extracellular RNA, whose function depends on the geometry of source, carrier and recipient.

High-priority ncRNAs should be perturbed before substantial pathology, during established amyloid accumulation and after tau-associated neurodegeneration or cognitive dysfunction emerges. miR-155 and miR-34a already show why single-stage experiments can mislead (4, 5, 19). Longitudinal human sampling should mirror this design to distinguish early drivers from compensation and late maladaptation.

### 10.2 Genetics, aging, sex, and human causal models

Human microglial xenografts show that APOE and triggering receptor expressed on myeloid cells 2 (TREM2) reshape responses to Aβ and favor distinct cell states (112), while APOE4 is linked to a damaging lipid-droplet microglial program (113). ncRNA mechanisms that converge on TREM2, lipid metabolism, phagocytosis or inflammatory signaling—including circ-Epc1/TREM2—should therefore be tested across relevant genetic backgrounds (101).

Aging alters mitochondrial competence, lysosomal function, chromatin state and RNA processing, whereas sex shapes immune and metabolic responses. Sex-dependent miRNA control of microglial fatty-acid synthesis shows that these variables can modify ncRNA biology (16). Human multimodal datasets should define age- and sex-dependent baselines rather than treating them as demographic covariates (96).

Patient-derived iPSC microglia, human microglial xenografts and ex vivo human microglia provide complementary forms of human causal evidence (43, 97, 112). No single platform recreates the aged human brain; confidence should come from triangulation across models.

### 10.3 Multimodal perturbation and quantitative RNA communication

A dedicated AD ncRNA atlas will require single-nucleus transcriptomics, small-RNA sequencing, long-read profiling, chromatin accessibility, proteomics and spatial pathology (96, 97). Multi-omics discovery such as the miR-28a-5p–SerpinA3N study provides a starting point (21), but the decisive advance will be perturbational: systematically manipulate candidate ncRNAs and measure the resulting cell-state transitions at single-cell resolution.

EV biology must also become quantitative. Studies should measure RNA copies per vesicle, the fraction of vesicles carrying the candidate, productive uptake and delivered abundance relative to the endogenous RNA pool (9, 10, 76). Because stoichiometric requirements differ between RISC-mediated repression and amplified endosomal TLR signaling, including TLR7 and, in human reporter systems, TLR8 (11), quantitative communication biology is essential for assigning mechanism.

### 10.4 Biomarker-guided stratification and research roadmap

Disease biomarkers classify AD, state biomarkers report active neuroimmune programs and pharmacodynamic biomarkers indicate target engagement. ncRNAs may add most value in the latter two roles, complementing rather than competing with established Aβ and p-tau assays. Human intervention studies (87) and actionable ncRNAs (12) show how state measurement can connect target biology to treatment monitoring.

Response to ncRNA therapy is likely to depend on genotype, stage, age, sex, inflammatory state, vascular or metabolic comorbidity and baseline RNA abundance. Precision trials should therefore connect biomarker-defined state to patient selection, RNA intervention, target engagement, state transition and clinical response (98, 110).

Future programs should integrate disease-relevant human cell states, causal ncRNA perturbation, genetic and disease-stage context, precision delivery and pharmacodynamic biomarkers. Therapeutic development should focus on pathological state transitions that can be modified through RNA biology rather than expression changes alone.

## 11 Discussion

The evidence now supports a shift from viewing ncRNAs as isolated inflammatory modifiers to treating them as regulators of neuroimmune state and intercellular communication. This conceptual advance is nevertheless uneven across the field. The strongest mechanistic evidence remains concentrated in miRNAs, microglia and Aβ-related biology, whereas lncRNAs, circRNAs, other small-RNA classes, astrocytic and vascular programs, and tau-responsive immune mechanisms are less mature. This asymmetry should not be interpreted as biological exclusivity. Microglia are the best-resolved ncRNA compartment in AD, but neuronal stress, astrocytic homeostasis, barrier function and peripheral immunity continuously reshape the same inflammatory environment (1–3, 30, 43, 73, 74).

Context dependence is therefore a defining property of ncRNA biology rather than a secondary source of experimental variability. miR-155 illustrates the trade-off most clearly: microglia-specific deletion can reduce amyloid pathology while destabilizing neuronal network function, whereas the same RNA also supports IFNγ-associated state transitions and phagocytic programs (4, 5). miR-146a spans inflammatory feedback, EV-associated tolerance and metabolic dysfunction (31, 82, 83). For extracellular miR-29a-5p, human TLR7/8 activation is supported by receptor-reporter assays, whereas the downstream neuroinflammatory effects in murine microglia and *in vivo* were demonstrated primarily as TLR7-dependent (11, 75). miR-25802 shows genuinely discordant directions across two substantially different systems: microglial/*in vivo* AD models in which endogenous miR-25802 promotes KLF4–NF-κB-dependent pathology, and an Aβ-exposed SH-SY5Y *in vitro* model in which milk-derived sEV delivery of miR-25802 is protective (6, 93). Recipient cell, carrier, dose and disease context are plausible modifiers, but this explanation remains untested. These observations argue against assigning ncRNAs fixed protective or pathogenic identities while also cautioning against overinterpreting cross-model discordance as proof of a specific context-switch mechanism.

The same distinction is essential when weighing evidence quality. Mechanistic closure and human relevance frequently do not coincide. circDlg1 combines a resolved RNA-protein mechanism with human disease-associated material (7), whereas patient-derived EV studies and AD iPSC microglia add functional testing to human-associated systems (9, 10, 43). By contrast, much of the wider literature still relies on acute Aβ exposure, immortalized cells, transgenic models or peripheral biomarker associations. Human atlases and ex vivo microglial datasets now provide the cellular resolution needed to identify disease-relevant states, but remain predominantly cross-sectional and descriptive (96, 97). A major unresolved issue is whether candidate ncRNAs initiate pathological state transitions, stabilize them once established, or arise as compensatory responses. Longitudinal and perturbational human-relevant studies are required to distinguish these possibilities.

Therapeutic translation exposes a second tension: the pleiotropy that makes ncRNAs attractive also narrows their therapeutic window. Direct inhibition of miR-485-3p demonstrates that one RNA intervention can improve Aβ, tau, inflammation and cognition (12), while microglia-targeted lipid nanoparticles, the corrected engineered-circular-RNA platform, and EV-based approaches show that increasingly sophisticated delivery systems can be coupled to ncRNA biology (13, 99–109). Yet target validation and delivery feasibility remain distinct: CNS exposure/BBB penetration, target-cell specificity, productive intracellular release, molecular target engagement, off-target network effects and innate RNA sensing each require direct evidence (11, 98–100). Therapeutic development should therefore prioritize controllable state reprogramming rather than maximal target suppression, with dose, disease stage, cell specificity, pharmacodynamic monitoring and delivery-specific safety defined early in development.

These considerations also refine the likely clinical role of ncRNA biomarkers. Their greatest value may not be as standalone diagnostic markers competing with established Aβ and phosphorylated-tau assays, but as readouts of neuroimmune state and target engagement. CSF, patient-derived EVs and intervention-responsive circulating RNAs already provide proof of principle for this role (9, 10, 12, 87, 94, 95). Linking such signatures to spatially and genetically defined cell states could support biologically informed patient stratification, particularly because APOE, TREM2, aging and sex alter the neuroimmune landscape in which a candidate ncRNA acts (16, 96, 111–113).

The field should therefore advance by narrowing, rather than expanding, its candidate list. Priority should be given to ncRNAs for which cell-state specificity, molecular mechanism, human convergence, therapeutic direction and measurable target engagement can be defined within the same biological framework. Such prioritization would move ncRNA research beyond catalogs of disease-associated expression and toward direct testing of whether specific RNA-regulated state transitions are both causally important and therapeutically controllable.

## 12 Conclusion

ncRNAs occupy a regulatory layer linking innate immune sensing, cell-state transitions, intercellular communication and the pathological feedback networks of AD. Three conclusions are supported most consistently: ncRNA effects are context dependent rather than intrinsically pro- or anti-inflammatory; intracellular and extracellular localization can fundamentally change mechanism; and ncRNAs can influence several disease domains simultaneously by tuning how neuroimmune, proteostatic and metabolic processes reinforce one another.

The translational opportunity lies in identifying a restricted set of human-supported ncRNA nodes whose function can be modulated with cell, compartment, dose and disease-stage precision. Progress will depend less on discovering additional differentially expressed RNAs than on establishing causal state control, quantitative intercellular RNA biology, productive CNS delivery and pharmacodynamic evidence of target engagement. Within this framework, ncRNAs may become not only biomarkers or therapeutic targets, but tools for selectively reprogramming the neuroimmune states that shape the response of the aging brain to Alzheimer pathology.
